# Impact of Single and Combined Salinity and High-Temperature Stresses on Agro-Physiological, Biochemical, and Transcriptional Responses in Rice and Stress-Release

**DOI:** 10.3390/plants11040501

**Published:** 2022-02-12

**Authors:** Lutfun Nahar, Murat Aycan, Shigeru Hanamata, Marouane Baslam, Toshiaki Mitsui

**Affiliations:** 1 Department of Life and Food Sciences, Graduate School of Science and Technology, Niigata University, Niigata 950-2181, Japan; f19m504g@mail.cc.niigata-u.ac.jp; 2 Department of Agricultural Botany, Sher-e-Bangla Agricultural University, Dhaka 1207, Bangladesh; 3 Laboratory of Biochemistry, Faculty of Agriculture, Niigata University, Niigata 950-2181, Japan; murataycan@agr.niigata-u.ac.jp (M.A.); hanamata@agr.niigata-u.ac.jp (S.H.)

**Keywords:** heat, salt stress, individual stress, coupled stress, independent/interactive effects, recovery

## Abstract

Here, for the first time, we aimed to identify in rice the key mechanisms and processes underlying tolerance to high-temperature (HT) or salt stress (SS) alone, the co-occurrence of both stresses, and recovery using physiological and biochemical measurements and gene expression analysis. We also investigated whether recovery from the two stressors depended on the relative intensities/relief of each stressor. Wild type (‘Yukinkomai’) rice plants were found to be more susceptible to salinity or heat applied individually. SS leads to a depletion of cellular water content, higher accumulation of Na^+^, and alterations in photosynthetic pigments. The stress-tolerant cultivar ‘YNU31-2-4’ (YNU) displayed a lower Na^+^/K^+^ ratio, higher water content in cells and improved photosynthetic traits, antioxidant system, and expression of defence genes. Strikingly, the SS + HT combination provided a significant level of protection to rice plants from the effects of SS alone. The expression pattern of a selected set of genes showed a specific response and dedicated pathways in plants subjected to each of the different stresses, while other genes were explicitly activated when the stresses were combined. Aquaporin genes were activated by SS, while stress-related (*P5CS*, *MSD1*, *HSPs*, and ions transporters) genes were shaped by HT. Hierarchical clustering and principal component analyses showed that several traits exhibited a gradually aggravating effect as plants were exposed to the combined stresses and identified heat as a mitigating factor, clearly separating heat + salt-stressed from salt-non-heat-stressed plants. Furthermore, seedling recovery was far more dependent on the relative intensities of stressors and cultivars, demonstrating the influence of one stressor over another upon stress-release. Taken together, our data show the uniqueness and complexity of the physiological and molecular network modules used by rice plants to respond to single and combined stresses and recovery.

## 1. Introduction

Rice is a staple food for over half the world’s population and is the main source of calories in developing countries [1]. Excessive salinity and high temperature are two of the most damaging ecological stress factors on rice growth and productivity worldwide [2]. Therefore, the production of major crops needs to increase dramatically—potentially by as much as 60–100% by 2050—to meet the growing demand [3]. In order to improve crop yield and meet future food demand, plant stress tolerance must be improved, and extensive efforts are needed to better understand the mechanisms underlying plant responses to simultaneous/sequential exposure to different (a)biotic stresses. To date, most studies on plant stress response have focused on the responses to a single stress condition, such as heat, drought, salinity, nutrients, microbes and herbivores [4,5,6,7,8]. Under field conditions, these stresses frequently occur together, resulting in massive damage to crop yields [9]. Only recently have different combinations of stresses been included in the research of plant stress response in situations mimicking natural conditions. However, these studies have mainly focused on a single time point and provide only a snapshot of general or specific changes induced by combined stresses.

Studies on combined stresses have revealed that plants act differently and evoke distinct integrated signal networks, which could be different from responses to the individual stresses, especially when stress factors have antagonistic interactions [5,10,11]. The outcome of combined or sequential stress can either be neutral, additive, synergistic or sometimes may lead to novel unpredictable responses [10,12]. However, common pathways and plant responses are also shared by some stress combinations. Within these signaling pathways and depending on the environmental stimuli, a range of physiological, morphological, or molecular reactions may occur. Temperature increases alter the major biological processes of plants by modifying the efficiency of enzymatic activities [13], thereby creating metabolic imbalance [14] and ultimately affecting plant growth and development [15]. Salinity induces Na^+^ toxicity, which can alter macro- and micro-nutrient uptake by root cells [16] and causes a seriously deleterious effect on genes and enzymes, resulting in metabolic deficiencies [17,18,19]. Stress-induced transcriptional changes—usually specific subsets of genes—are orchestrated by transcription factors and genes that act together to mediate various stress responses [20,21]. Salt stress inhibits the activities of the ion transporters or antiporters located in the plasma membrane [22]. The alteration of ion channels in response to salt and heat exposure is essential for stress tolerance. High-affinity K transporters (HKT) and sodium/potassium antiporters (NHX) enhance salinity tolerance by regulating Na^+^ in plant tissues [23]. The HKT1 and HKT2 subfamilies are K^+^ transporters and play a significant role in maintaining the Na^+^/K^+^ ratio on the plasma membrane [24]. NHXs are important proteins activated to reduce salt toxicity at the whole plant level [25]. These fine-tuning strategies allow plants to respond to environmental challenges and coordinate/balance these responses with developmental programs that serve different growth, defense, reproduction, and survival functions.

Here, we first evaluated the responses to both single and combined HT and SS conditions in two rice genotypes with contrasting stress tolerance levels and assessed how both stressors interact/differ on the traits related to plant growth and development. Our study design aimed to identify the physiological, biochemical, and key transcript responses that were consistently associated with stress tolerance to inform the future development of stress-tolerant rice cultivars. Then, we sought to elaborate on the mechanisms underlying rice resistance to individual and combined heat and salinity stresses. Moreover, we aimed to highlight the pace of post-stress recovery dynamics linking physiological stress impacts to recovery rate. We hypothesized that (1) the rice genotype showing single stress tolerance might not show the same tolerance to combined stresses; (2) responses to the two stresses might share common physiological processes and other mechanisms and might have a negative or additive impact on growth and physiology, and a more specific response on rice than a single stress, or similar response to a single stress when one of the single stress played a predominant role on rice; and (3) interconnectedness of plants response to heat or saline environments through multiple physiological, metabolic, and molecular mechanisms.

## 2. Results

### 2.1. Survival, Growth, and Morpho-Physiological Responses of Rice Subjected to Single and Combined Heat and Salinity Stresses

In this study, we evaluated heat stress (HT) or salinity (SS) alone, the co-occurrence (SS + HT) of both stresses, and recovery by the comparative analysis of tolerant (YNU; ‘YNU31-2-4’) and susceptible (WT; ‘Yukinkomai’) cultivars. YNU was developed by the precise introgression of the *hitomebore salt tolerant 1* (*hst1*) gene through a single nucleotide polymorphism (SNP) marker-assisted selection (MAS) technique from “Kaijin” [26,27].

**Figure 1:**
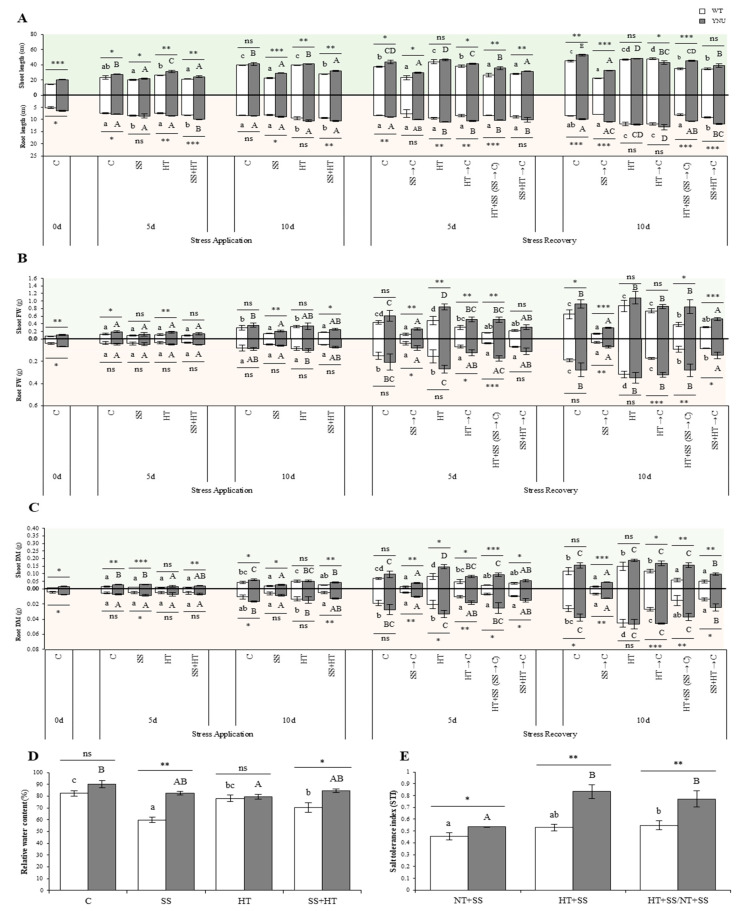
Morphological responses to salinity (SS), high-temperature (HT), and combined salinity and heat (SS + HT) of ‘Yukinkomai’ (WT) and ‘YNU31-2-4’ (YNU) rice seedlings; (**A**) Shoot (top panel) and root (lower panel) lengths, (**B**) shoot (top panel) and root (lower panel) fresh weight (FW), (**C**) shoot (top panel) and root (lower panel) dry matter (DM), (**D**) relative water content (RWT), and (**E**) salt tolerance index (STI) of WT and YNU plants under control normal-temperature (NT) (C: 26/23 °C, 0 mM NaCl), salt stress (SS: 26/23 °C, 75 mM NaCl), high-temperature (HT: 30/26 °C, 0 mM NaCl), combined stress (SS + HT: 30/26 °C, 75 mM NaCl), and stress-release (recovery) conditions. Bars = 10 cm. Values represent the means ± SE determined from three independent experiments using 4 plants in each experiment. Means within the same graph followed by different lowercase (WT) and uppercase (YNU) letters are significantly different at *p* < 0.05 according to the Tukey’s HSD test. Asterisks represent statistical significance of difference using Student’s *t*-statistic with *** *p* < 0.001, ** *p* < 0.01, * *p* < 0.05, ns: non-significant.

The effect of heat and salinity stress exposure was different, with salinity being the treatment that most affected the growth of shoot and roots in both cultivars (Figure S1 and Figure 1A–C). Shoot and root length values of plants grown under HT were very similar to those obtained under normal temperature (NT) conditions. Compared with the heat and control treatments, the SS + HT treatment reduced shoots fresh weight (FW) and dry matter (DM) of both cultivars after a short exposure (5 day-after-treatment; DAT5), while, surprisingly, there was no phenotypic difference at DAT10 compared to control unstressed plants (Figure 1B,C). Salinity was the treatment that most affected the growth of wild type (WT) plant shoots, through rolled and yellowish leaves and roots, whereas those of YNU remained flat and stayed green even at 75 mM NaCl. Further, 75 mM NaCl under NT reduced the survival of WT seedlings by 100%, whereas all seedlings of YNU survived (Figure S1). The YNU cultivar showed faster recovery rates of growth traits than WT. The plants, mainly WT, showed impaired recovery from salinity (SS → C), and they developed non-reversible tissue damage (Figure 1A–C), persistent leaf damage and desiccation (Figure S1). Unlike salt stress, unexpectedly, continuous HT stress (10 d recovery) resulted in a positive increase (+70%) in root FW in both cultivars (Figure 1B, lower panel).

Under single and combined stress, the YNU plants maintained significantly higher leaf relative water content (RWC) than WT. Indeed, as with the YNU seedlings, the RWC of WT seedlings declined significantly by 27% with the single SS (Figure 1D). However, compared with SS, the decline in this parameter was overall greater under HT and the HT + SS combination (Figure 1D). RWC reduction was less impacted, with no significant differences, in YNU when the stresses applied were SS or SS + HT (*p* > 0.05^ns^; Figure 1D).

Under NT conditions, the salt tolerance index (STI) of YNU was higher than WT (Figure 1E). A shift to HT conditions enhanced STI by ca. 16%, although this was not statistically significant, while YNU showed ca. 57% higher values on this index than under NT. The combined impact of salinity and heat conditions increased STI. The two factors, elevated temperature and elevated salinity, again had a cumulative impact on the STI.

**Figure 2:**
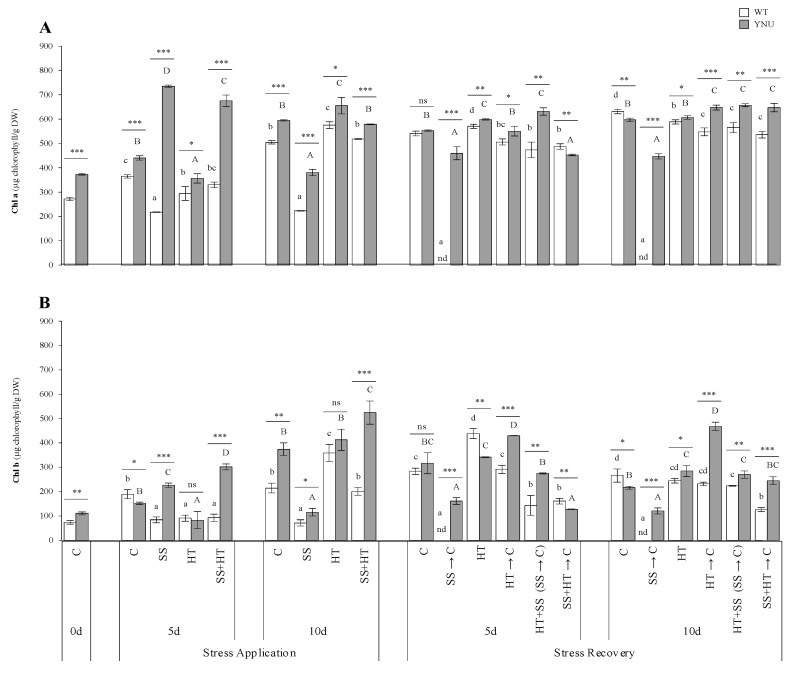
Chlorophyll content of rice seedlings under salinity (SS), high-temperature (HT), and combined salinity and heat (SS + HT) conditions; (**A**) Chlorophyll a (Chl a) and (**B**) chlorophyll b (Chl b) content of WT and YNU plants under control (C: 26/23 °C, 0 mM NaCl), salt stress (SS: 26/23 °C, 75 mM NaCl), high-temperature (HT: 30/26 °C, 0 mM NaCl), and combine stress (SS + HT: 30/26 °C, 75 mM NaCl) conditions. Values represent the means ± SE determined from three independent experiments using 4 plants in each experiment. Means within the same graph followed by different lowercase (WT) and uppercase (YNU) letters are significantly different at *p* < 0.05 according to the Tukey’s HSD test. Asterisks represent statistical significance of difference using Student’s *t*-statistic with *** *p* < 0.001, ** *p* < 0.01, * *p* < 0.05, ns: non-significant.

In addition, leaf chlorophyll (Chl) content was found to be higher in the YNU cultivar than WT (Figure 2). Chlorophyll levels were significantly decreased after DAT5 in WT under SS and HT alone and in YNU exposed to SS alone at DAT10, but not under HT alone and the SS + HT for both cultivars. Our results showed that SS was the treatment that most affected plants, as it resulted in the largest reduction in Chl a and b. With respect to HT alone or SS + HT, salinity had a strong negative effect on the photosynthetic pigments, resulting in a significant and progressive reduction of these over the time of exposure to the stress. This reduction extended as far as WT death after DAT10. YNU restored the chlorophyll pigment better than the C treatment and WT, especially in the presence of HT (HT → C) and HT-related treatments (HT + SS (SS → C) and SS + HT → C) (Figure 2). The recovery strategy of the WT was not effective under SS alone, which led to non-reversible plant losses at DAT15 and DAT20, while this cultivar showed a set of stress-recovery strategies under HT alone or relief from SS alone under continuous HT (HT + SS (SS → C)).

### 2.2. Na^+^ and K^+^ Content in Shoots and Roots under Individual and Combined Salt and Heat Stresses

**Figure 3:**
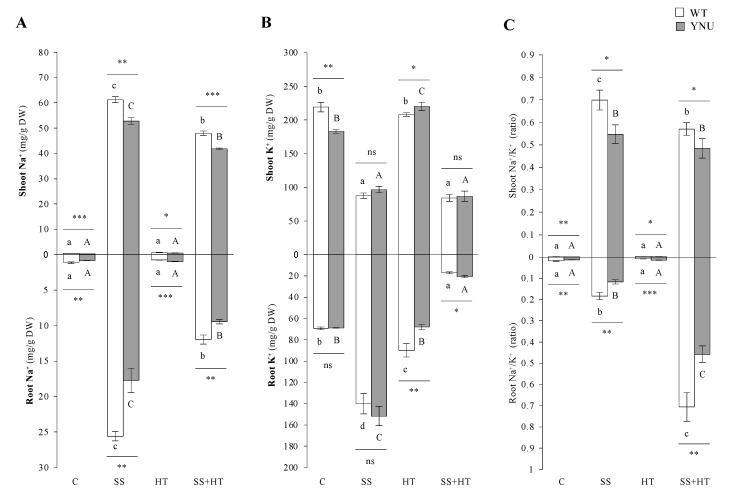
Na^+^ and K^+^ ion content of rice seedlings under salinity (SS), high-temperature (HT), and combined salinity and heat (SS + HT) conditions; (**A**) K^+^ content of shoot (top panel) and root (down panel), (**B**) Na^+^ content of shoot (Top panel) and root (down panel), and (**C**) Na^+^/K^+^ ratio of shoot (Top panel) and root (down panel) of WT and YNU seedlings under control (C: 26/23 °C, 0 mM NaCl), saline (SS: 26/23 °C, 75 mM NaCl), high-temperature (HT: 30/26 °C, 0 mM NaCl), and combined salinity and heat (SS + HT: 30/26 °C, 75 mM NaCl) conditions. Values represent the means ± SE determined from three independent experiments using four plants in each experiment. Means within the same graph followed by different lowercase (WT) and uppercase (YNU) letters are significantly different at *p* < 0.05 according to Tukey’s HSD test. Asterisks represent statistical significance of difference using Student’s *t*-statistic with *** *p* < 0.001, ** *p* < 0.01, * *p* < 0.05, ns: non-significant.

As expected, Na^+^ concentrations in control and HT alone treatments remained similarly low and were insignificant as these plants did not receive additional NaCl. SS alone was the stress that most increased Na^+^ concentrations (115× in WT vs. 250× in YNU in shoots and 23× in WT vs. 21× in YNU in roots) relative to plants grown under controlled conditions (Figure 3A). Na^+^ concentrations in shoots and roots of both cultivars grown under the SS + HT treatment was ca. 22% lower than those found in plants grown under SS. Under control conditions, shoot K^+^ concentrations were significantly lower in YNU shoots than in WT (Figure 3B), whereas under SS, a significant reduction in K^+^ concentrations were found in leaves–ca. 60% and 47% in the control shoots in WT and YNU, respectively (Figure 3B). K^+^ concentrations found in rice grown under C conditions and HT were very similar, and no significant variations were found. Under SS + HT, a significant reduction in K^+^ concentrations were found in roots, with this being the treatment that most reduced K^+^ concentrations of all the treatments analyzed (Figure 3B). These changes in Na^+^ and K^+^ content resulted in a pronounced increase in the Na^+^/K^+^ ratio in rice shoots grown under SS alone. This ratio did not differ significantly between the C and HT conditions in both shoots and roots (Figure 3C). Under salinity, the ratio was 291× and 497× in WT and YNU, respectively, their C levels in the shoot (Figure 3C). The Na^+^/K^+^ ratio was reduced in the shoot by HT when it was combined with SS, 18% lower in WT and 11% lower in YNU in SS + HT compared with SS. In contrast, this ratio was much higher in roots under SS + HT than with salinity or heat alone.

### 2.3. Oxidative Stress, Osmoprotectant Accumulation, and Antioxidant

**Figure 4:**
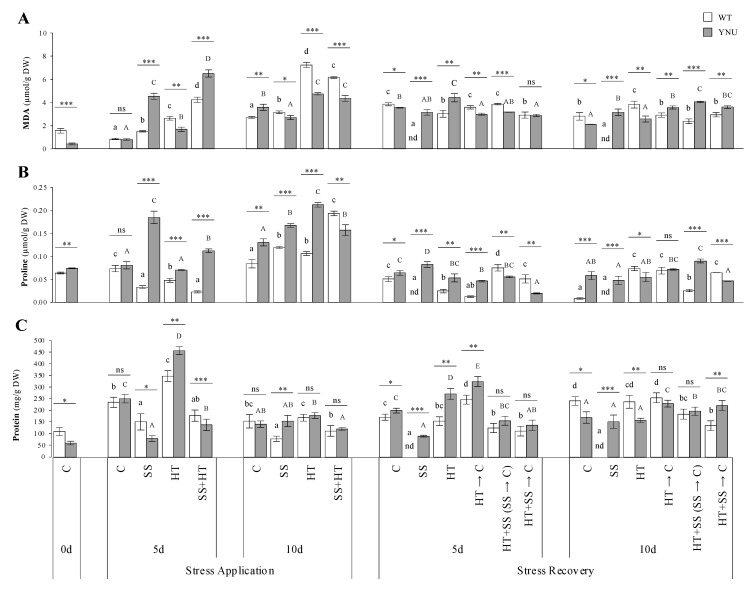
Biochemical responses to salinity (SS), high-temperature (HT), and combined salinity and heat (SS + HT) of rice seedlings; (**A**) Malondialdehyde (MDA), (**B**) proline, and (**C**) protein of WT and YNU plants under control (C: 26/23 °C, 0 mM NaCl), salt stress (SS: 26/23 °C, 75 mM NaCl), high-temperature (HT: 30/26 °C, 0 mM NaCl), and combined salinity and heat (SS + HT: 30/26 °C, 75 mM NaCl) conditions. Values represent the means ± SE determined from three independent experiments using four plants in each experiment. Means within the same graph followed by different lowercase (WT) and uppercase (YNU) letters are significantly different at *p* < 0.05 according to Tukey’s HSD test. Asterisks represent statistical significance of difference using Student’s *t*-statistic with *** *p* < 0.001, ** *p* < 0.01, * *p* < 0.05, ns: non-significant. nd: not determined.

**Figure 5:**
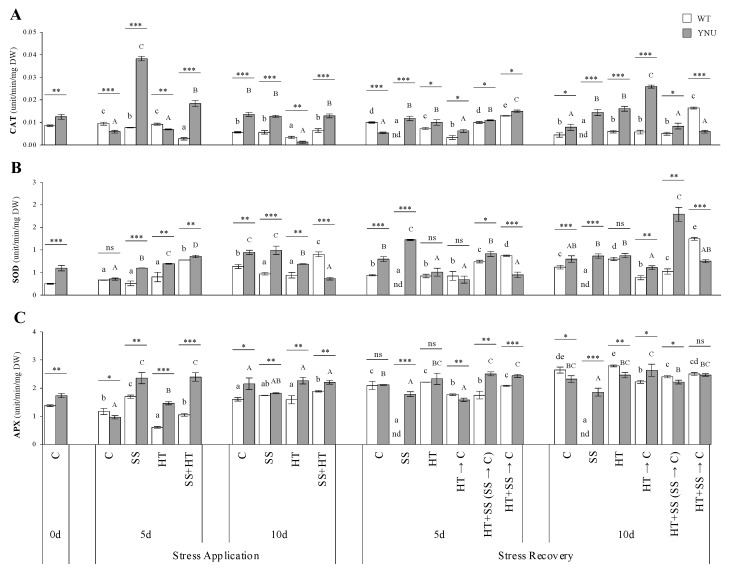
Antioxidant enzyme responses to salinity (SS), high-temperature (HT), and combined salinity and heat (SS + HT) of rice seedlings; (**A**) catalase (CAT), (**B**) superoxide dismutase (SOD), and (**C**) ascorbate peroxidase (APX) of WT and YNU plants under control (C: 26/23 °C, 0 mM NaCl), salt stress (SS: 26/23 °C, 75 mM NaCl), high-temperature (HT: 30/26 °C, 0 mM NaCl), and combined salinity and heat (SS + HT: 30/26 °C, 75 mM NaCl) conditions. Values represent the means ± SE determined from three independent experiments using four plants in each experiment. Means within the same graph followed by different lowercase (WT) and uppercase (YNU) letters are significantly different at *p* < 0.05 according to Tukey’s HSD test. Asterisks represent statistical significance of difference using Student’s *t*-statistic with *** *p* < 0.001, ** *p* < 0.01, * *p* < 0.05, ns: non-significant. nd: not determined.

In the absence of stress, the levels of malondialdehyde (MDA), proteins, and enzymes in the two cultivars were lower than the other treatments, while in the presence of stress, the overall levels of these parameters were either similar or higher than C (Figure 4 and Figure 5). At DAT5, YNU plants grown under SS and the SS + HT combination showed the highest MDA values (Figure 4A), while this increase in WT was not pronounced under salinity as with combined stress. After long-term exposure to stresses, DAT10, MDA concentrations were lower in both cultivars under SS, while in the HT and SS + HT treatments they were increased, mainly in WT, where they were ca 3× higher than in control plants (Figure 4A). MDA levels were reversed upon stress release in the recovery, thereby rebuilding lipid peroxidation and damaged tissue. Concentrations of the osmoprotectant proline, proteins, and main enzyme activities increased under the SS and SS + HT conditions, in parallel with the MDA increases, after 5 days of stress exposure (Figure 4B,C and Figure 5A–C), while the WT showed the highest levels of proline after DAT10 when stresses were applied individually or combined (Figure 4B). During stress, protein concentration accumulated significantly under HT stress after DAT5 in both cultivars (Figure 4C), whereas the SS alone or combined with heat (SS + HT) significantly decreased this parameter (Figure 4C).

Under HT conditions, only the catalase (CAT) enzyme had a similar or decreased activity with respect to C plants during stress (Figure 5A). The coupled stress induced the synthesis of superoxide dismutase (SOD) in both cultivars after short-term exposure (Figure 5B). In contrast, the single stresses did not change SOD activity in the two phases of the stress occurrence in WT and DAT10 in YNU (Figure 5B). As for ascorbate peroxidase (APX), except for the increase in YNU under SS and/or the HT treatments at DAT5, the different stress treatments and post-stress recovery resulted in a similar pattern throughout the experiment (Figure 5C). For prolonged periods after stress-release, DAT15 and 20, YNU maintained higher protein content and CAT activity and similar proline, while WT leaf proline levels were continuously higher than in the C condition (Figure 4 and Figure 5).

### 2.4. Single and Combined Heat and Salinity Stresses Cause Significant Reprograming of Gene Expression Profiles

**Figure 6:**
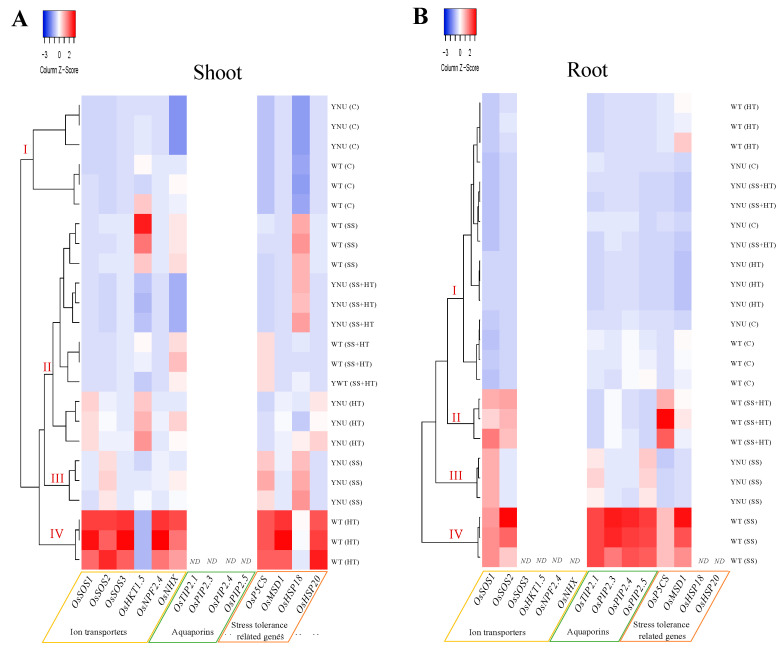
Hierarchical clustering analysis (HCA) of the relative transcript level of ion transporters, aquaporins, and stress-related genes in (**A**) shoot and (**B**) root tissues of WT and YNU seedlings under control (C: 26/23 °C, 0 mM NaCl), salt stress (SS: 26/23 °C, 75 mM NaCl), high-temperature (HT: 30/26 °C, 0 mM NaCl), and combined salinity and heat (SS + HT: 30/26 °C, 75 mM NaCl) conditions. Kendall’s Tau and Average linkage were used as distance measurement and clustering methods, respectively. ND: not detected.

The morpho-physiological and biochemical analysis clearly revealed abiotic stress induced responses in both cultivars following 10 days of treatment and therefore this was chosen as a time point to analyze the selected set of genes in rice leaves and roots. This is based upon the hypothesis that at this time point, the severity of the stress and the underlying physiological processes could determine if stress impacts are quickly reversible or if tolerance or permanent damage occurs, indicating susceptibility. We selected the most significantly stress-regulated genes from each stress condition [28,29,30,31,32,33,34,35,36,37]. As a result, rice cultivars have different interaction pathways, either neutral, additive, or synergistic, while responding to single and combined stress conditions. The expression of these genes was different depending on the stress applied and plant tissue involved. In shoots, under HT, an up-regulation of small heat shock proteins (*OsHSP20*), stress-related (*OsMSD1* and *OsP5CS*), and ion transporter (salt overly sensitive family; *OsSOS1*, high-affinity K^+^ transporter; *OsHKT1;5*, nitrate transporter; *OsNPF2;4*, and Na^+^/H^+^ antiporter; *OsNHX*) genes was observed in WT (Figure 6A and Figure S2). Salinity alone induced the activation of *OsP5CS*, *OsSOS1*, and *OsHSP18* in YNU. Under SS + HT, an activation of *OsHSP18* in YNU and *OsNHX* and *OsP5CS* in WT was observed (Figure 6A and Figures S2–S4). In contrast, this combined stress down-regulated the K^+^ transporter *OsHKT1;5* and Na^+^/H^+^ antiporter *OsNHX* genes in the salt-tolerant YNU cultivar. These results are not comparable to those found in the below-ground tissue when these stresses were applied. In roots, SS-treated WT presented the highest values of aquaporins, stress-related, and ion transporter genes (Figure 6B and Figures S2–S4). SS + HT treatment showed similar ion transporter and stress-related gene expression. On the other hand, in YNU exposed to individual SS up-regulation of *OsSOS1* and, to a lesser extent, the tonoplast intrinsic protein (*OsTIP2;4*) and plasma membrane intrinsic protein (*OsPIP2;5*) gene expression was observed (Figure 6B and Figure S3). In contrast to salinity, the HT alone in both cultivars and combined stresses in YNU led to a down-regulation of the genes studied (Figure 6B). The heat map derived from a one-way hierarchical clustering analysis (HCA) grouped the rice shoots grown for either the individual or the combination of the two stresses into four major clusters; (I) rice plants under the C treatment (II) WT and YNU subjected to a single SS or HT, respectively, and the combined stress for both cultivars, (III) SS-treated YNU, and (IV) heat-treated WT plants (Figure 6A). Roots also were grouped into four groups consisting of; (i) C and HT alone in both cultivars and YNU subjected to the combined SS + HT, (ii) combined HT + SS stress in WT, (iii) SS alone in WT, and (iv) WT exposed to salt stress alone.

### 2.5. PCA

**Figure 7:**
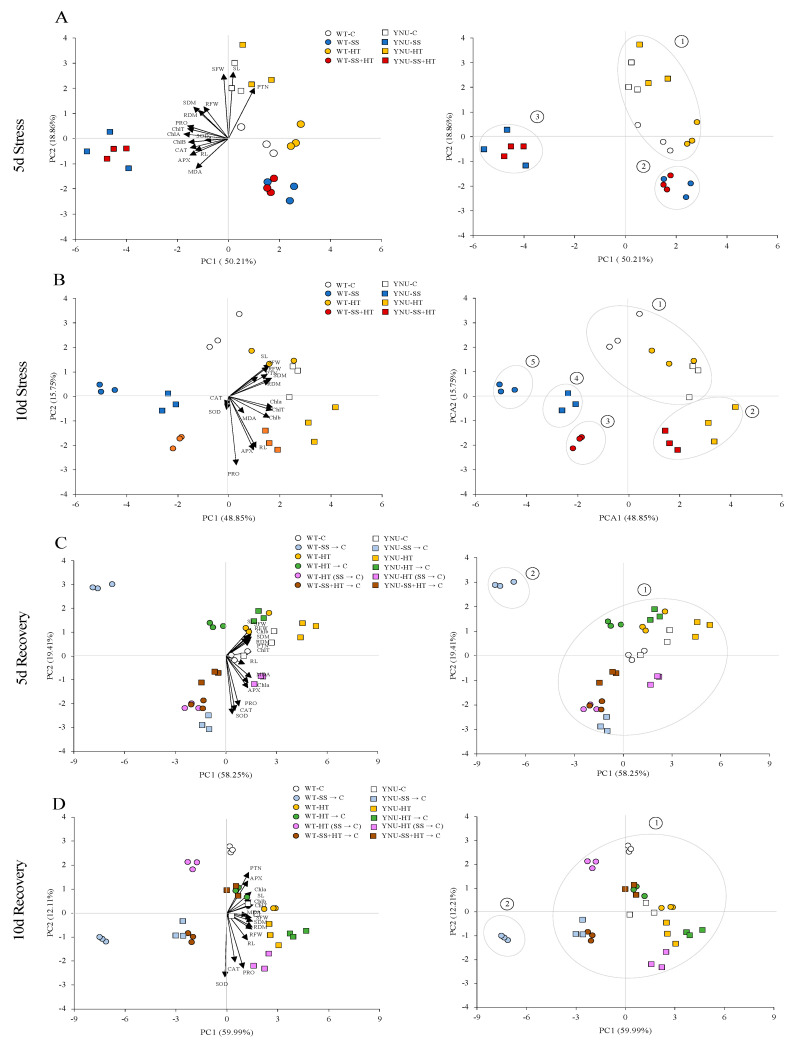
Principal component analyses (PCA) of WT and YNU traits under (**A**) DAT5; 5 days of stress, (**B**) DAT10; 10 days of stress, (**C**) DAT15; 5-day stress-release recovery, and (**D**) DAT20; 10-day stress-release recovery.

The PCA showed that in the treatments of both cultivars, WT (square symbol) and YNU (rectangular symbol) and traits were associated with PC1 and PC2 (65~78%), of which PC1 was the major component (49~60%) (Figure 7, Tables S2–S5). Five days of exposure to stresses grouped the C and HT-treated rice into one group (group 1) associated with shoot FW and shoot length, while the SS alone treatment or the combination with HT clearly separated the two cultivars into 2 groups; group 2 (WT) and group 3 (salt-tolerant YNU). This latter group showed higher growth, photosynthetic-related pigments and antioxidants/osmoprotectants (Figure 7A). Stress severity (DAT10) separated the rice cultivars and treatments into five clusters: two groups positively correlated with PC1, namely, group 1 including controls and HT-treated WT and group 2 harboring YNU under HT alone or combined with SS. Both groups were positively correlated with most of the traits studied. WT under the SS + HT was in group 3 associated with higher enzyme activity. Interestingly, SS led to both cultivars being clearly isolated into two separate groups, specifically group 4 containing YNU and higher intensity WT (group5) (Figure 7B). Under stress-recovery (DAT15 and DAT20), except for the SS-treated WT (group 2), rice plants were able to repair and grow following single and combined stress release (group1).

Among the 5-day stress traits, under SS conditions, WT and YNU were clearly separated from each other, but the C and HT traits were found close together after a 10-day stress exposure. At this time point the distance between traits and cultivars was more clearly seen, especially since the distance between traits and cultivars SS and SS + HT traits were well separated. Additionally, the distinction that was not seen between control and HT in the first 5 days of exposure was seen much more clearly on the 10th day (Figure 7B). All traits of the recovery period (5 days) were clearly separated, with the most significant separation being between WT and YNU in the SS → C application. HT stress exposed traits localized near the C groups in both WT and YNU plants, with the HT → C application showing the same pattern. HT (SS → C), SS + HT → C traits in WT and SS → C traits in YNU were localized near to each other (Figure 7C). The distance between traits became smaller after the 10-day recovery period. The SS → C traits in YNU and SS + HT → C traits in WT were observed close to each other. The greatest distance was measured between WT and YNU in the HT (SS → C) application. Distances between the HT → C, HT, and C traits were found to be small. Traits related to the exposure to HT were found at the same positions in the WT and YNU cultivars (Figure 7D).

## 3. Discussion

Salt stress and heat are two of the widespread co-occurring environmental conditions for the plants grown in arid and semi-arid zones. With global warming and climate change, understanding how plants respond to simultaneous heat and salinity stress exposure is crucial for sustainable agriculture. However, until recently, studies have investigated plant responses to these stresses individually [38,39,40], and such studies do not allow the mechanisms plants will use to respond to combined stresses to be predicted. Furthermore, seedling growth could be a potentially helpful trait for early screening against heat and salt stress. In this paper, we elaborate on the dynamics of the physiological and biochemical changes and regulatory mechanisms underlying rice responses to individual and combined heat and salinity, and the acclimatory/repair phase.

The accumulated impact of coupled stress had a minimal effect on rice growth and survival (Figure 1A–E and Figure S1) compared to controls. We found that stress from SS can cause severe disruptions to growth and many of the physiological processes involved in the establishment of rice seedlings, albeit the magnitude of the impact on WT was generally greater. Indeed, the growth penalty of rice plants to salinity or heat applied individually was coherent with previous studies [27,41]. In contrast, rice grown under SS + HT exhibited above- and below-ground growth (shoot/root DW and shoot/root length) that was higher than that observed in the salinity condition, although it was similar to the heat treatment. Biomass was positively related to nitrogen concentration as well as K^+^/Na^+^ ratio under single stress; nevertheless, these correlations between growth and physiological parameters were weaker under combined stress [42,43]. This complex mode of correlation between growth and physiological traits under this stress combination might be due to different, sometimes even opposing, signaling pathways induced by combined stress [43]. Alexieva et al. [44] showed that an RWC decline was the main factor causing growth decrease as a response to osmotic stress, which can be induced also by salinity and heat. Under SS, in our study, sensitive WT was more affected by the decline in RWC than the salt-tolerant YNU cultivar (Figure 1D). This suggests that both cultivars had a similar sensitivity when subjected to HT and combined stress. Leaf RWC is an essential indicator of the balance between water supply to the leaf tissue and transpiration rate. The decrease in the water potential in both stresses, especially SS, may result in reduced cell growth and root/shoot growth and causes inhibition of cell expansion and compositional changes in the cell wall [45]. Salinity and heat affect the regular metabolism of the cell, such as the C-reduction cycle, light reactions, energy charge, and proton pumping and lead to the production of ROS molecules.

The high-salt availability and heat impair the process of photosynthesis by disturbing the photosynthetic pigments [46,47,48], reducing photosystem II activity [48], and inducing changes in the performance of important enzymes, including RuBP and ROS scavengers [47]. As a result, HT alone or combined with SS increased Chl a and b. Camejo et al. [48] revealed that an increased chlorophyll a/b ratio was observed along with a significant decrease in the chlorophyll to carotenoid ratio in heat-tolerant tomato cultivars and sugarcane plants. This shows that a change in the ratio of the pigments may play a role in acquiring thermotolerance against heat shock [49]. Similarly, short-term (DAT5) exposure to salinity induced chlorophyll synthesis, then (DAT10 and recovery) caused more accelerated degradation of chlorophyll a and b in rice leaves. The positive response in the short-term might be due to the temporary maintenance of the photosynthetic machinery since the Rubisco enzyme concentration remains relatively stable because it has a half-life of several days [50]. However, the stress intensity leads to a rapid decrease in the abundance of Rubisco small subunit (*rbcS*) transcripts in rice, indicating decreased synthesis [51]. Reduced accumulation of chlorophyll in rice plants, especially the salt-sensitive WT, may be due to the decreased biosynthesis of Chl attributed to the deactivation of various enzymes (i.e., 5-aminolevulinate dehydratase) [52] and/or its increased degradation. Additionally, the salinity-induced stomatal closure—in response to the reduced leaf water potential—in order to avoid water loss through transpiration, checks CO_2_ intake, which leads to oxidative damage and no assimilation, thereby leading to chlorophyll decline. The reduced RWC observed in salt-treated plants causes shrinkage of the cell, and later as a consequence, the cellular material becomes more viscous, which leads to denaturation of proteins [50]. Rivero et al. [2] data showed that when SS and HT were applied, the photosynthetic parameters, including pigments, were almost identical to those found in heat-treated plants. New evidence documents the maintenance of a high transpiration rate in response to HT stress, which points to a potential major physiological trade-off between the need for heat escape and the need for water conservation to protect photosystems dictated by increasing evaporative demand [53,54,55]. Our findings suggest that when salinity is applied together with heat, the latter confers some advantages to overcoming salinity alone. This may be achieved by increasing the transpiration rate and diminishing the damage to the photochemical activity of PSII and the photosynthetic machinery, increasing CO_2_ uptake and diffusion through the open stomata for photosynthesis and therefore photoassimilate availability for rice growth with respect to salt exposure alone.

It has been postulated that the cellular Na^+^/K^+^ ratio is an indicator of cell toxicity. As expected, Na^+^ content and the Na^+^/K^+^ ratios were higher in salt-treated shoots and roots than in controls, while the K^+^ content decreased in shoots in both stresses. Strikingly, the coupled SS + HT stress displayed a lower accumulation of Na^+^ in both tissue parts and the Na^+^/K^+^ ratio in leaves compared with SS, which can be seen as an evolved avoidance mechanism in rice to protect the photosynthetic machinery in the leaves. Importantly, K^+^ uptake was markedly reduced by the combination SS + HT relative to the single stresses and close to that of control plants. This reduction in the K^+^ influx observed in rice roots exposed to the coupled stresses might be partly due to an increase in the K^+^ efflux, but this remains to be confirmed. For instance, under HT and SS, the Kout channel GORK-mediated K^+^ efflux activated by ROS was upregulated in roots [56,57]. GORK could trigger PCD (programmed cell death) and/or prevent activation of anabolic enzymes by K^+^, thereby releasing energy for stress adaptation and repair [58]. Cuin and Shabala [59] showed that ROS could promote K^+^ efflux in root epidermal cells, which was significantly reduced by the exogenous application of several osmoprotectants, including proline. Other mechanisms triggering changes in K^+^ uptake from the soil, K^+^ transport and accumulation throughout the plant, and stomatal regulation under salt and heat could involve post-translational regulatory networks—involving phosphorylation/dephosphorylation, modifications in targeting, and interactions with regulatory partner proteins—in different plant organs [60]. Furthermore, it can be speculated that the regulation of transport and distribution of ions in different plant parts may play a critical role under combined stress as a physiological adaptation strategy to cope with excessive amounts of Na^+^ and Cl^−^. This result is important as it shows that the coupled impacts of independent stressors could act synergistically and increase stressor tolerance. Na^+^ content and the Na^+^/K^+^ ratio in leaves and roots were different between WT and YNU and therefore may be linked to the mechanism related to salt tolerance or susceptibility. An overload of Na^+^, as observed in WT plants, can dramatically depolarize the plasma membrane, leading to K^+^ efflux via depolarization-activated outward-rectifying K^+^ channels [61]. This ion nutrition imbalance disturbs efficient stomatal regulation, which results in a depression of photosynthesis and growth [62]. As the YNU harbors the *hst1* (loss-of-function in *OsRR22*) gene, this primarily led to the upregulation of *OsHKT1;1* (encoding a high-affinity K^+^ transporter) that functions as a Na^+^ transporter contributing to the salt resistance of the *hst1* mutant.

Membranes are the primary site of ion-specific salt and/or heat injury. At DAT5, SS was the single treatment that caused the highest lipid peroxidation levels (Figure 4A). The coupled stress led to damaging effects stronger than any of the individual stresses. After the stress treatment, the plants recovered the control values, but the salinity dose intensified the negative impact on WT plant growth, leading to death. It is worth noting that in YNU the induction of antioxidant systems and repair mechanisms occurred at an earlier time point, while outside the double treatments, it was the HT stress that had the most pronounced damaging effects upon 10 days of stress in WT. These data raise the possibility that at the 10-day time point of stress, WT plants were in an acute stress phase, while the YNU may have overcome the oxidative stress due to the rapid response and entered an acclimation phase. In addition, YNU possesses efficient mechanisms for Na^+^ exclusion from the cytosol and thus may not require a high level of antioxidant activity at DAT10, as this cultivar simply does not allow excessive or lower ROS production and lipid peroxidation, in the first instance. These data show in more detail the dynamics of the biochemical changes to fully resolve stress and acclimatory phases. SS alone and the combined SS + HT showed the highest osmoprotectant proline accumulation (Figure 4B). In this sense, proline has been suggested, in different plants, to be a singlet oxygen and superoxide quencher during osmotic stress as it could reduce ROS damage, including lipid peroxidation [63]. Proline can also have broader functions connected with photosynthesis and energy regulation [64,65]. Under SS, proline accumulation resulted from the activation of its synthesis at the transcriptional level through *P5CS* activity (Figure 6A and Figure S4A). However, in heat-treated WT, *P5CS* transcripts were up-regulated while the proline accumulation was inhibited probably due to interruption at the post-transcriptional level and/or concurrent inhibition of its degradation. Similarly, Rivero et al. [2] observed that tomato plants grown under SS preferentially accumulated proline, through *P5CS* and *P5CR* activities, whereas HT and the coupled SS + HT treatment led to an accumulation of glycine betaine (GB) and did not favor proline due to the strong induction of the enzymes implicated in proline degradation.

Following higher lipid peroxidation, exposure to salinity and double stress enhanced the antioxidant defense in YNU either enzymatically (e.g., SOD, CAT, APX) or non-enzymatically (proline) (Figure 4 and Figure 5). Maintenance of higher levels of antioxidants in YNU under salinity could be a good strategy used by the plants to counter the negative effects of ROS. Increased antioxidant activity in YNU should be treated as a trait directly conferring salinity stress tolerance and survival, while WT not displaying such an increase died. As the increase in detoxifying enzyme activities i.e., peroxidases and the formation of ROS, occurs in the tissue itself, cross-linking of cell wall components might strengthen the mechanical properties of the wall to better withstand the changes in turgor pressure of osmotic stress [66,67,68]. A high antioxidant capacity of halophytes vs. glycophytes has been suggested as one of the important reasons for the greater ability of the former to tolerate extreme environmental changes, including high-salinity levels [69]. Foyer et al. [70] reported that photosynthetic limitation is often accompanied by ROS accumulation in the thylakoid stroma, which can be detoxified by the action of plastid localized SOD and APX. More specifically, cytosolic ascorbate peroxidase 1 (APX1) was identified as a key player protein in response to combined stress in Arabidopsis, pointing to the importance of oxidative stress under these conditions [71].

In the light of the above patterns that go some way to untangling the interactions between different stress treatments, we clustered the data on agro-physiological and biochemical traits, and we obtained three clusters at DAT5 (1; control + HT-treated plants, 2; WT under SS alone and combined stresses, and 3; YNU plants) that further separated, with the dominant effect of salinity over heat, into five clusters at DAT10 that pinpoint candidates (i.e. proline, APX) of the abiotic stress response of potential interest for engineering stress-tolerant plants (Figure 7). This pattern suggests an antagonistic interaction between the individual salinity and heat treatments. The coupled stress combinations show that SS does not change the patterns of the traits in the salt-tolerant YNU induced by the individual heat treatments (Figure 7B). Additionally, results revealed that the extent of recovery would be dependent on each stressor and combination of stressors. Contrary to our expectations, WT and YNU seedlings that received the combination of stresses had a comparable recovery to those that initially experienced individual stress. Additionally, at the whole-plant level, there were minimal differences in growth between seedlings that were and were not relieved from salinity (Figure 1 and Figure S1). Differences in recovery across treatments (and cultivars) can be governed by several intrinsic physiological mechanisms that could be explained by cell water content-inducing short-term stomatal closure and CO_2_ uptake (gas exchange rates) because growth is more sensitive than photosynthetic C assimilation to heat and salinity, thus providing additional substrate for growth. Second, greater osmoprotectant accumulation makes the magnitude of recovery greater and more evident. Third, early signaling pathway activation and gene regulation induce acclimation mechanisms. It is noteworthy that the physiological/biochemical differences (and clusters) in the SS + HT may explain the opposing gene expressions, in which both stresses might reprogram the different gene expression patterns observed individually or collectively.

To understand the transcriptional changes displayed in rice acclimations to single and combined stresses, we analyzed the patterns of selected genes under stress. Notably, WT leaves produced under HT tended to specifically up-regulate defense-related genes transcripts, i.e., *OsP5CS*, *OsMSD1*, HSPs, and SOS family members. Our results showed that the *OsHSP18* and *OsHSP20* genes were specifically expressed in leaf tissues. *OsHSP18* was induced not only under HT stress but also under SS + HT in both WT and YNU. Li et al. [72] and Zhang et al. [73] detected increased numbers of several proteins identified as HSPs under combined stress in poplars and *Carissa spinarum*. Additionally, it has been shown that HSPs accumulate under combined stress both at the transcript and proteome levels [74,75,76], emphasizing a conserved protective response between single independent stresses and their combination in cereals, grasses, and dicots [77]. Involvement of heat response pathways in the acclimation of plants to salt stress has been previously suggested by the up-regulation of HSPs and HSFs in response to both SS and HT [78,79]. Furthermore, a considerable increase in HSP level has been found under combined stress than with separately applied stress [80,81,82,83]. These results correspond to the basic role of HSPs/chaperone activity in the protein turnover during stress condition and their major protective role against protein aggregations [83,84]. Salinity led to distinct gene expression signatures involving ion transporter-related genes, suggesting that every single stress is unique in its effects on plant metabolism, physiology, and survival, and requires a unique gene expression response for plant adaptation. Indeed, such transporters for ion exclusion most probably involve the orchestrated action of several complementary mechanisms, including overexpression of SOS-mediated Na^+^ exclusion from the cell and efficient control of aquaporin channels in the cell-cell pathway. On the other hand, the combination has revealed overexpression of SOS family members and *OsP5CS* in the root, with an overlap of these transcripts in response to salinity alone, indicating that SS responses might dominate the acclimation response of rice to the stress combination. The result of phosphorylated SOS1 is improved Na^+^ efflux, thus reducing Na^+^ toxicity [85]. This protein is essential for the cell-level regulation of Na^+^ efflux, and its overexpression was related to salt tolerance [86]. Long-distance Na^+^ transportation from root to shoot is also facilitated. PIPs family members are hypothesized to target the plasma membrane [33], especially the root, and are involved in salt stress responses [36].

## 4. Materials and Methods

### 4.1. Plant Material, Experimental Design, and Stress Treatment and Recovery Scheme

In this study, we evaluated HT or SS alone, the co-occurrence (SS + HT) of both stresses, and recovery by the comparative analysis of tolerant and susceptible cultivars. The cultivars were selected based on our previous research reporting abiotic stress tolerance. ‘Yukinkomai’ (WT) was used as the salt-susceptible cultivar [27] and has a high yield potential of 6.84 t ha^−1^ [87]. ‘YNU31-2-4’ (YNU) was used as the salt-tolerant cultivar [39], which was developed by the precise introgression of the *hitomebore salt tolerant 1* (*hst1*) gene through a single nucleotide polymorphism (SNP) marker-assisted selection (MAS) technique from “Kaijin” [26,27]. The causative SNP conferring the high salinity tolerance of the *hst1* mutant line corresponded to the third exon of the *Os06g0183100* gene, which is predicted to encode a B-type response regulator designated *OsRR22*.

Rice seeds were de-husked, surface sterilized, and washed with a 2% sodium hypochlorite solution containing 0.02% Tween 20. The seeds were placed on 1% agar plates containing 1⁄2 Murashige and Skoog (1⁄2 MS) medium at pH 5.8 and incubated in dark conditions at 30 °C. Three days after germination (DAG3), the plates were transferred to a controlled growth chamber: 26/23 °C day/night, 12-h light cycle, 350 µmol m^−2^ s^−1^ light intensity, and 70% relative humidity for 4 days. At DAG7, healthy and uniform looking seedlings were transferred to a hydroponic system by placing them into holes (1 plant/hole) on a Styrofoam seedling float device, and the emerging radicle was carefully inserted through the nylon mesh. The Styrofoam device was suspended on a tray filled with Yoshida solution at pH 5.0 [88]. Next, 35–40 seedlings of each genotype were placed side-by-side on the same device, and each treatment was repeated using four biological replicates for a total of 280–320 seedlings per treatment and per genotype.

The salt and/or heat stresses were imposed on 14-day-old rice seedlings by supplementing Yoshida’s medium with 75 mM NaCl and/or increasing temperature (30/26 °C day/night).

Seedlings from the different genotypes were then subjected to the following individual and coupled treatments for 10 days after treatment (DAT10) application: C (control, Yoshida solution, 0 mM NaCl, 26/23 °C), SS under normal temperature (salt stress, Yoshida solution, 75 mM NaCl, 26/23 °C), HT (high-temperature, Yoshida solution, 0 mM NaCl, 30/26 °C), and SS + HT (salt stress + high temperature stress, Yoshida solution, 75 mM NaCl, 30/26 °C) (Table 1).

In order to assess recovery from salinity under non-heated (SS → C) and heat conditions, no NaCl was applied to the Yoshida solution after DAT10, and part of the blocks was selected to receive relief from the HT treatments (fully recovery; SS + HT → C) while the other block had sustained HT (half-recovery; HT + SS (SS → C)) (Table 1). Relief from HT was applied by restoring the normal temperature. One block underwent the sustained HT treatments (HT).

All treatments were arranged in a completely randomized design. Measurements and samplings were carried out after 5 and 10 days of stress treatment (DAT5 and DAT10), and a 5- and 10-day stress-release (recovery) period (DAT15 and DAT20). Shoots and roots were harvested and immediately frozen in liquid nitrogen. Tissue was ground into fine powder in the presence of liquid nitrogen and then kept at −80 °C until use. Frozen samples were used for RNA extraction and biochemical and antioxidant enzyme activity assays. A separate sub-sample of the leaf and root materials was freeze-dried and stored at room temperature for Na^+^ and K^+^ determination. All experiments were performed in biological triplicates.

### 4.2. Growth Assessment and Na^+^ and K^+^ Measurement

Plant height and root length were measured on DAT5~20. Shoot and root fresh weights (FW) were measured by cutting the seedling from the cotyledonary node. The shoot and root dry weight (DW) were measured after drying the shoot and root in an oven at 85 °C until the weight remained constant.

The salinity tolerance index (STI) under normal (SS) and high (SS + HT) temperature was determined as STI = (FW_salt stress seedlings_/FW_control seedling_) × 100 (%)

Sodium (Na^+^) and potassium (K^+^) ions in shoots and roots were quantified by a wet digestion method [89]. Dried, finely powered plant samples (10 mg) were digested in HNO_3_ solution. Samples were incubated in a heat bath at 60 °C for 2 h. After cooling, H_2_O_2_ was added to the digest and heated again at a range of 60 to 120 °C. The digested solution was shaken gently and filtered through 0.2-µm filters (Whatman, Maidstone, England), and the solid fraction was discarded. The contents of Na^+^ and K^+^ in the extract were quantified by Polarized Zeeman Atomic Absorption spectrophotometry (Z-6100, Hitachi, Tokyo, Japan).

### 4.3. Measurement of Relative Water Content and Chlorophyll Concentration

Relative water content (RWC). The FW of the leaflet was immediately measured after cutting. Then, the leaflet was immersed in H_2_O and incubated at normal room temperature. After 4 h, the leaflet was taken out, thoroughly wiped to remove the water on the surface and weighed to obtain turgid weight (TW). Then, the leaflet was put in a drying oven for 24 h and weighed to obtain DW. RWC = [(FW − DW)/(TW − DW)] × 100 (%).

Chlorophyll content. Pigment content was determined using the method of Hori et al. [90]. Chlorophyll a (Chl a) and chlorophyll b (Chl b) contents per DW were calculated by the formula:

Chl a = (16.5 × A665 − 8.3 × A650) × volume (V)/weight (W)

Chl b = (33.8 × A650 − 8.3 × A665) × V/W

### 4.4. Measurement of Malondialdehyde, Proline, Protein, and Antioxidant Enzyme Activities

Lipid peroxidation was measured by determining the amount of MDA as Dhindsa and Matowe [91] with the modifications listed by Mestre et al. [92]. The MDA concentration was calculated using an extinction coefficient for MDA of 155 mM^−1^ cm^−1^.

Free proline content was measured by a colorimetric assay as described by Bates et al. [93], with slight modifications. Proline was extracted with 2 mL toluene, incubated for 30 min, and absorbance was read at 520 nm.

Frozen leaf powder subsamples (20 mg) were homogenized in a cold mortar with 2 mL of 50 mM phosphate buffer (pH 7) containing 0.1 mM of Na-EDTA. The homogenate was centrifuged at 20,000× *g* for 10 min at 4 °C, and the supernatant was used to measure protein concentration and antioxidant enzyme activities [94].

Protein concentration was measured by the Bradford assay [95].

Superoxide dismutase (SOD, EC 1.15.1.1) activity was assayed using the method of Cakmak and Marschner [96]. One unit of SOD activity was defined as the amount of enzyme leading to 50% inhibition of nitro blue tetrazolium (NBT) reduction at 25 °C.

Catalase (CAT, EC 1.11.1.6) activity was measured by monitoring the consumption of H_2_O_2_ substrate at 240 nm for 3 min [97]. The reaction mixture consisted of 100 μL of enzyme extract, potassium phosphate buffer (50 mM, pH 7.6), 0.1 mM EDTA, and 20 mM H_2_O_2_ in a 2 mL volume.

Ascorbate peroxidase (APX, EC 1.11.1.11) activity was assayed as a decrease in absorbance at 290 nm for 1 min according to the method of Amako et al. [98]. The assay solution contained 100 μL of extract sample, 50 mM potassium phosphate buffer (pH 7.6), 0.5 mM H_2_O_2_, and 0.1 mM ascorbate. The reaction was initiated by adding the enzyme extract, and the decrease in absorbance was recorded.

### 4.5. RNA Extraction and cDNA Synthesis

The total RNA was extracted from shoot and root at DAT10 (10 days of stress exposure). Total RNA was extracted using the TRizol method [99] according to the manufacturer’s protocol (Invitrogen, CA, USA). RNA integrity was analysed by gel electrophoresis, and its concentration was measured using a NanoDrop ONEC spectrophotometer (Thermo Fisher Scientific, USA). The cDNA templates were generated from the total RNA samples by reverse transcription using ReverTra Ace^®^ qPCR RT Master Mix with gDNA Remover (Toyobo, Osaka, Japan).

### 4.6. Real-Time Quantitative PCR Analysis

Real-time PCR analyses were performed using the CFX96 Real-Time PCR Detection System (Bio-Rad Laboratories GmbH, CA, USA). RT-PCR amplifications were performed in a 10 µL reaction volume mixture containing 1 µL of cDNA, 3.6 µL H_2_O, 0.2 µL of 10 pmol forward (sense) primer, 0.2 µL of 10 pmol reverse (antisense) primer, and 5 µL SsoFast^TM^ EvaGreen^®^ Supermix (Bio-Rad Laboratories GmbH, CA, USA). Each reaction for each gene was performed in triplicate following PCR protocol: 2 min 98 °C, 2 s 98 °C, 5 s 65 °C, 10 s 75 to 95 °C for melting curve (39–50 cycles). The primer sets and reference gene (*OsUBQ5*) used for RT-PCR are shown in Table S1, and their specificity was measured by separating the PCR product on 0.7% agar gels. The relative expression levels of genes were calculated by using the 2^−∆∆CT^ method as proposed by Livak and Schmittgen [100]. Control (0 mM NaCl, 26/23 °C (day/night)) at 0d has been used as a reference sample for calculating the gene relative quantification.

### 4.7. Statistical and Multivariate Analyses

Physiological and biochemical measurements and the qPCR assays were analyzed statistically using R software (V3.6.1, https://www.r-project.org, accessed on 20 December 2021). Means separation was determined using Tukey’s honestly significant difference (HSD) test at *p* < 0.05 with R software, including the ‘glht’ function in the ‘multcomp’ package as reported by Hothorn et al. [101]. Principal component analysis (PCA), based on morpho-physiological and biochemical data values under stresses and the recovery period, was used to identify a minimal number of principal components that accounted for most of the variation in the traits. Associations between growth, physiological, and biochemical traits and first and second principal components (eigenvalues > 1) and among FC values within each treatment were studied using Pearson correlation analysis. Index values for each treatment were first established by assessing the response of SS, HT, SS + HT, and recovery compared to their control values, then the responses of all the traits under each treatment were combined and used as index values for the PCA analysis. Heat maps based on hierarchical clustering of gene expression variation were plotted using Ward’s method. Differences in mode probabilities across and between response patterns were determined with an χ2 test at *p* ≤ 0.05. The hierarchical cluster analysis (HCA) was executed on the correlation matrix of 2 cultivars and response variables of transcript levels in rice shoots and roots. Kendall’s Tau and Average linkage were used as distance measurement and clustering methods, respectively. PCA and HCA were created using R software.

## 5. Conclusions

In nature, variation in temperature and salinity are frequent environmental constraints restricting plant performance, and in the field, they can occur simultaneously or independently. Currently, plant breeders are engaged in a search for climate-resistant varieties. Studying and identifying the strategies used by rice subjected to combined stresses could elucidate the ways of importing the genes/pathways used by this plant into other cultivars or species to develop climate-resilient crops. The response of plants to each of the different stresses applied individually is unique and involves many transcripts and genes that are not altered in response to multifactorial stress combinations. This study, therefore, suggests an important role for Na^+^ and K^+^ uptake and transport, water limitation, osmoprotectant accumulation, and the expression of transcripts and ROS-scavenging enzymes responsible for the accumulation observed under a combination of stresses, which cannot be deduced from the independent response of plants to each of the single stresses. As a result, it becomes much clearer that different stresses could interact to minimize the negative impact on plant health and performance by coordinating the allocation of assimilates between plant growth and defense responses, even if the effect of each stress applied individually is strongly negative. While heat-treated WT induced intensive reprograming of antioxidant defense components and stress-responsive genes, genes coding for the ion transporter expression profile were overexpressed under salinity alone. In contrast, we found that these genes were almost all down-regulated in YNU, especially under heat stress, suggesting an energy investment in growth. The harmful impact of abiotic stresses should serve as a dire warning to our society and, hence, further studies addressing the impact of combinations of stresses on reproductive stages and yield could prove critical for our global food production and safety.

## Figures and Tables

**Table 1:** The scheme of the experimental design.

	Conditions	Temperature (Day/Night °C)	[Salt] (mM NaCl)
Stress Application	**C:** Control	26/23	0
	**SS:** salt stress (single stress)	26/23	75
	**HT:** high-temperature stress (single stress)	30/26	0
	**SS + HT:** combined SS + HT stresses	30/26	75
Recovery	**C**	26/23	0
	**SS → C:** recovery from SS	26/23	75 → 0
	**HT**	30/26	0
	**HT → C:** recovery from HT	30/26 → 26/23	0
	**HT + SS (SS → C):** recovery from SS alone	30/26	75 → 0
	**SS + HT → C:** fully recovery from the combined stresses	30/26 → 26/23	75 → 0

## Data Availability

Not applicable.

## Supplementary Materials

The following supporting information can be downloaded at: https://www.mdpi.com/article/10.3390/plants11040501/s1. Table S1. Primer list of the genes used in this study. Tables S2–S5. Loading values and percentage contribution of principal component (PC) variables. Figure S1. Picture at 5 and 10 days of ‘Yukinkomai’ (WT) and ‘YNU31-2-4’ (YNU) rice responses to salinity (SS), high-temperature (HT), combined salinity and heat (SS + HT) stress treatments, and the recovery stage. Figure S2. Relative expression level of ion transportation genes. Figure S3. Relative expression level of aquaporin genes. Figure S4. Relative expression level of stress-related genes.
