# Supplementary material for: Impact of Single and Combined Salinity and High-Temperature Stresses on Agro-Physiological, Biochemical, and Transcriptional Responses in Rice and Stress-Release
Source: Plants (Basel). 2022 Feb 12;11(4):501. doi: 10.3390/plants11040501 (PMC8876766; doi:10.3390/plants11040501)
Supplement: Supplementary file 1 [file plants-11-00501-s001.zip › Nahar et al_Supp Fig S1_Plants.pptx]

## Slide 1
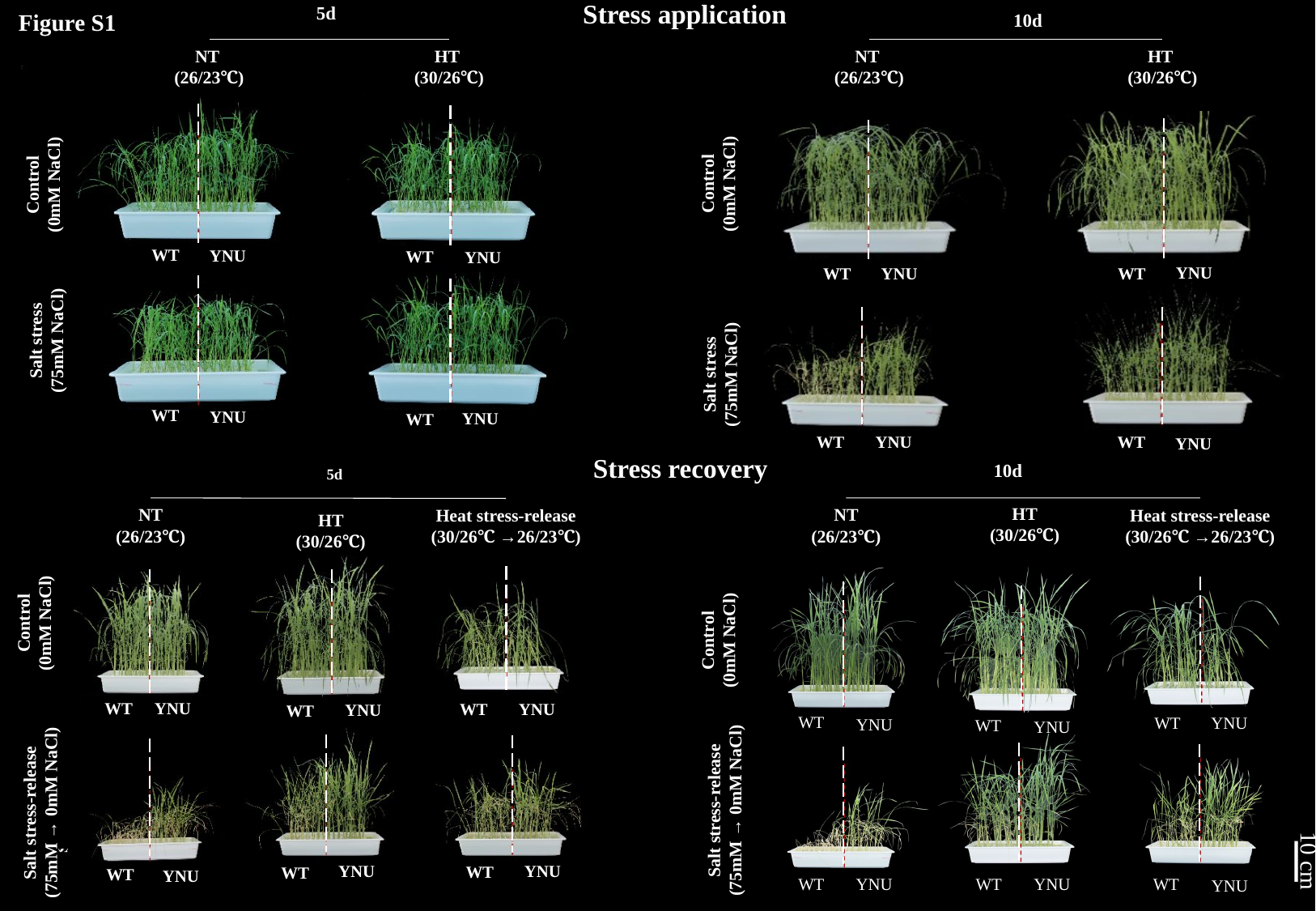

Stress application
5d
NT
(26/23℃)
HT
(30/26℃)
Control
(0mM NaCl)
WT
YNU
WT
YNU
Salt stress
(75mM NaCl)
WT
YNU
YNU
WT
10d
NT
(26/23℃)
HT
(30/26℃)
Control
(0mM NaCl)
YNU
WT
WT
YNU
Salt stress
(75mM NaCl)
WT
WT
YNU
YNU
Stress recovery
10d
5d
HT
(30/26℃)
NT
(26/23℃)
NT
(26/23℃)
Heat stress-release
(30/26℃ →26/23℃)
Heat stress-release
(30/26℃ →26/23℃)
HT
(30/26℃)
Control
(0mM NaCl)
Control
(0mM NaCl)
YNU
WT
YNU
WT
YNU
YNU
WT
WT
WT
YNU
YNU
WT
YNU
Salt stress-release
(75mM → 0mM NaCl)
Salt stress-release
(75mM → 0mM NaCl)
10 cm
YNU
YNU
WT
WT
WT
YNU
WT
WT
YNU
WT
YNU
YNU
Figure S1
