# Supplementary material for: Impact of Single and Combined Salinity and High-Temperature Stresses on Agro-Physiological, Biochemical, and Transcriptional Responses in Rice and Stress-Release
Source: Plants (Basel). 2022 Feb 12;11(4):501. doi: 10.3390/plants11040501 (PMC8876766; doi:10.3390/plants11040501)
Supplement: Supplementary file 1 [file plants-11-00501-s001.zip › Nahar et al_Supp Fig S2-4_Plants_MA.pptx]

## Slide 1
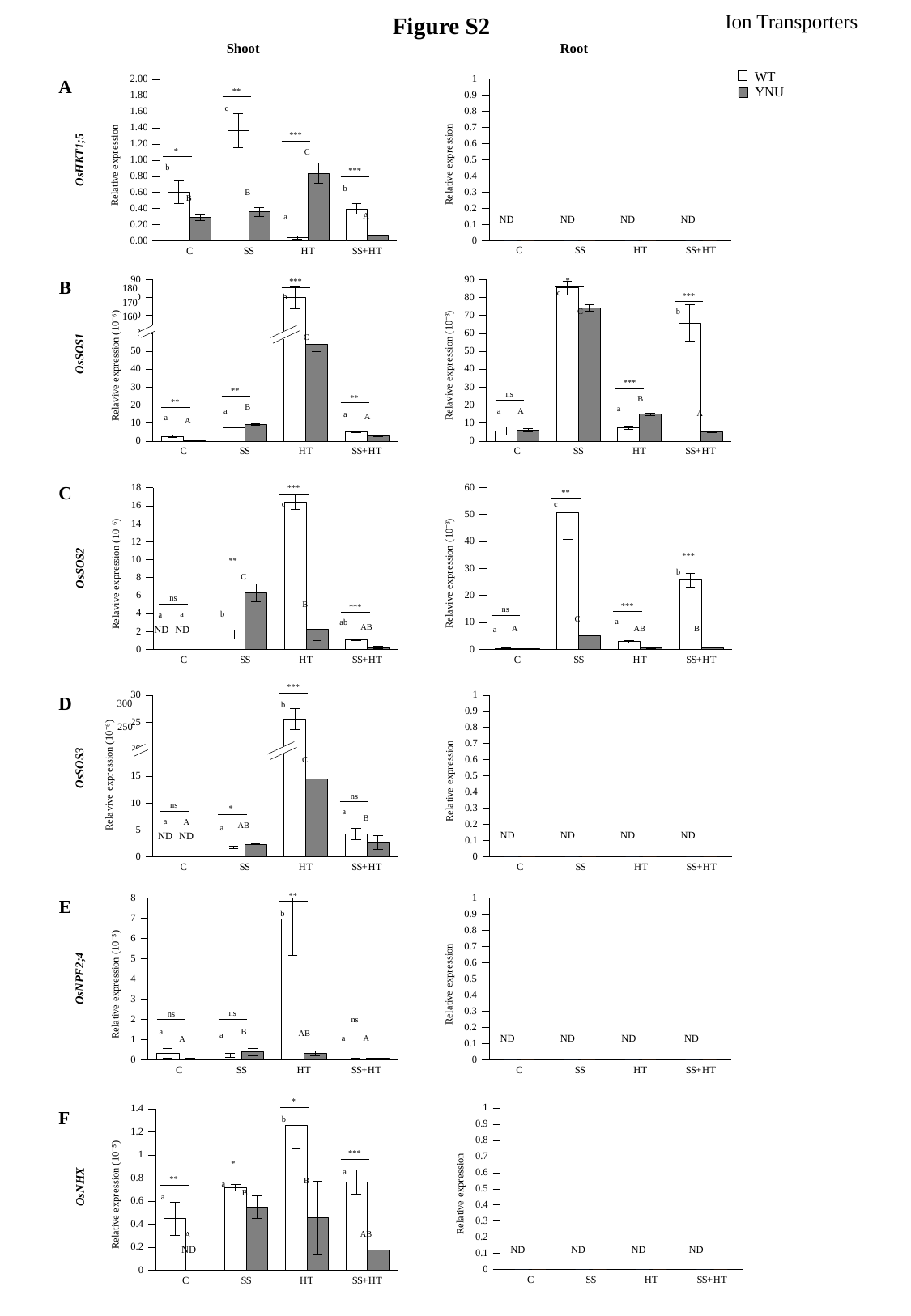

Ion Transporters
Figure S2
Shoot
Root
WT
YNU
A
### Chart
| Category | Yukinkomai | YNU |
|---|---|---|
| C | 0.6077513146125918 | 0.2900406156213391 |
| SS | 1.365975508931288 | 0.35990384640738565 |
| HT | 0.04458224378974982 | 0.8397758831856494 |
| SS+HT | 0.3980544966115079 | 0.07140884782575924 |OsHKT1;5
c
C
b
b
B
B
A
a
**
***
*
***
### Chart
| Category | Yukinkomai | YNU |
|---|---|---|
| C | 0.0 | 0.0 |
| SS | 0.0 | 0.0 |
| HT | 0.0 | 0.0 |
| SS+HT | 0.0 | 0.0 |ND
ND
ND
ND
*
### Chart
| Category | Yukinkomai | YNU |
|---|---|---|
| C | 5.590671846488436 | 6.293849880229523 |
| SS | 85.35208574958737 | 74.09612881273858 |
| HT | 7.620156862361448 | 15.027142897414372 |
| SS+HT | 65.77385312093233 | 5.1897126452482585 |c
b
C
B
a
a
A
A
***
***
ns
B
***
### Chart
| Category | Yukinkomai | YNU |
|---|---|---|
| C | 2.740549775205268 | 0.16666666666666666 |
| SS | 7.407824519076413 | 9.401188382298711 |
| HT | 80.0 | 53.98312813062379 |
| SS+HT | 5.031958814259018 | 2.869788112536364 |180
b
170
160
C
OsSOS1
**
**
**
B
a
a
A
a
A
C
***
### Chart
| Category | Yukinkomai | YNU |
|---|---|---|
| C | 0.0 | 0.0 |
| SS | 1.6522340268153677 | 6.327543113405763 |
| HT | 16.45990938049268 | 2.276055734565981 |
| SS+HT | 1.05865931651822 | 0.1920079466233958 |OsSOS2
ND
c
**
C
ns
B
***
a
b
a
ab
AB
ND
### Chart
| Category | Yukinkomai | YNU |
|---|---|---|
| C | 0.23258298791326126 | 0.2250088363151006 |
| SS | 50.68989738126152 | 5.163466732809562 |
| HT | 2.991748737906288 | 0.48200813533931947 |
| SS+HT | 25.631300387413443 | 0.6208211009750657 |c
b
C
a
B
AB
A
a
**
***
***
ns
***
### Chart
| Category | Yukinkomai | YNU |
|---|---|---|
| C | 0.0 | 0.0 |
| SS | 1.7825092324632363 | 2.3101504101294563 |
| HT | 25.6 | 14.539652701509054 |
| SS+HT | 4.263308207554261 | 2.732203346951195 |300
250
OsSOS3
ND
ND
b
C
a
B
a
A
AB
a
ns
ns
*
D
### Chart
| Category | Yukinkomai | YNU |
|---|---|---|
| C | 0.0 | 0.0 |
| SS | 0.0 | 0.0 |
| HT | 0.0 | 0.0 |
| SS+HT | 0.0 | 0.0 |ND
ND
ND
ND
**
### Chart
| Category | Yukinkomai | YNU |
|---|---|---|
| C | 0.32499568882783786 | 0.056080620684338146 |
| SS | 0.23172496291522102 | 0.38759549961666134 |
| HT | 6.962873477640978 | 0.31869258034613945 |
| SS+HT | 0.042507876729093154 | 0.06556022335972504 |OsNPF2;4
b
a
B
AB
a
A
a
A
ns
ns
ns
E
### Chart
| Category | Yukinkomai | YNU |
|---|---|---|
| C | 0.0 | 0.0 |
| SS | 0.0 | 0.0 |
| HT | 0.0 | 0.0 |
| SS+HT | 0.0 | 0.0 |ND
ND
ND
ND
*
### Chart
| Category | Yukinkomai | YNU |
|---|---|---|
| C | 0.4463078006252792 | 0.0 |
| SS | 0.7152452797333706 | 0.5464532737158482 |
| HT | 1.2526705536725018 | 0.45427945316565016 |
| SS+HT | 0.7655458855474611 | 0.1772812099366473 |b
a
OsNHX
B
a
B
a
AB
A
ND
***
*
**
### Chart
| Category | Yukinkomai | YNU |
|---|---|---|
| C | 0.0 | 0.0 |
| SS | 0.0 | 0.0 |
| HT | 0.0 | 0.0 |
| SS+HT | 0.0 | 0.0 |F
ND
ND
ND
ND

## Slide 2
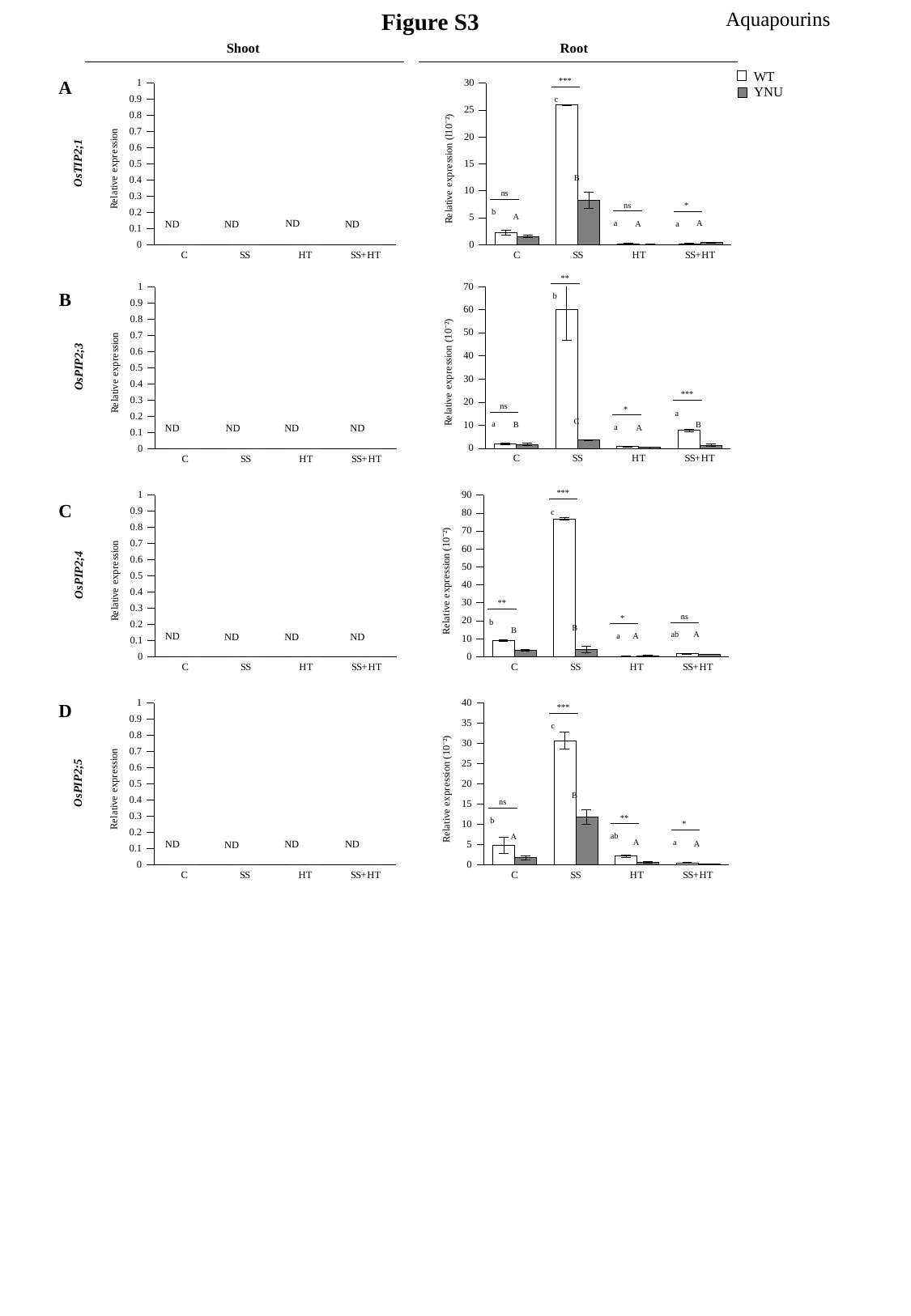

Aquapourins
Figure S3
Shoot
Root
WT
YNU
***
A
### Chart
| Category | Yukinkomai | YNU |
|---|---|---|
| C | 0.0 | 0.0 |
| SS | 0.0 | 0.0 |
| HT | 0.0 | 0.0 |
| SS+HT | 0.0 | 0.0 |OsTIP2;1
### Chart
| Category | Yukinkomai | YNU |
|---|---|---|
| C | 2.274895915908075 | 1.5819473479987127 |
| SS | 25.949496906902862 | 8.259488498145815 |
| HT | 0.19735123026236276 | 0.06463833219661082 |
| SS+HT | 0.1940172037968654 | 0.3818895560657225 |c
B
ns
ns
*
b
A
A
a
A
a
ND
ND
ND
ND
**
### Chart
| Category | Yukinkomai | YNU |
|---|---|---|
| C | 1.9636003296114906 | 1.7278554572834948 |
| SS | 60.07109660160097 | 3.5526874265614694 |
| HT | 0.7885099793602338 | 0.392864266209802 |
| SS+HT | 7.794890065593553 | 1.3951496055761634 |b
***
ns
*
a
C
a
B
B
a
A
### Chart
| Category | Yukinkomai | YNU |
|---|---|---|
| C | 0.0 | 0.0 |
| SS | 0.0 | 0.0 |
| HT | 0.0 | 0.0 |
| SS+HT | 0.0 | 0.0 |OsPIP2;3
ND
ND
ND
ND
B
***
### Chart
| Category | Yukinkomai | YNU |
|---|---|---|
| C | 8.985852290392343 | 3.5348633414223567 |
| SS | 76.84082642781745 | 4.166562132740368 |
| HT | 0.20985501676266974 | 0.5268101492811682 |
| SS+HT | 1.6542654824338545 | 1.32401881630594 |c
b
B
B
ab
A
a
A
**
ns
*
### Chart
| Category | Yukinkomai | YNU |
|---|---|---|
| C | 0.0 | 0.0 |
| SS | 0.0 | 0.0 |
| HT | 0.0 | 0.0 |
| SS+HT | 0.0 | 0.0 |OsPIP2;4
ND
ND
ND
ND
C
D
### Chart
| Category | Yukinkomai | YNU |
|---|---|---|
| C | 4.88089911046171 | 1.7206270389957388 |
| SS | 30.715952393814984 | 11.82962284815026 |
| HT | 2.111177277729944 | 0.5675078761542396 |
| SS+HT | 0.4268928509539352 | 0.16713763721462285 |***
c
B
ns
**
b
*
ab
A
A
a
A
### Chart
| Category | Yukinkomai | YNU |
|---|---|---|
| C | 0.0 | 0.0 |
| SS | 0.0 | 0.0 |
| HT | 0.0 | 0.0 |
| SS+HT | 0.0 | 0.0 |OsPIP2;5
ND
ND
ND
ND

## Slide 3
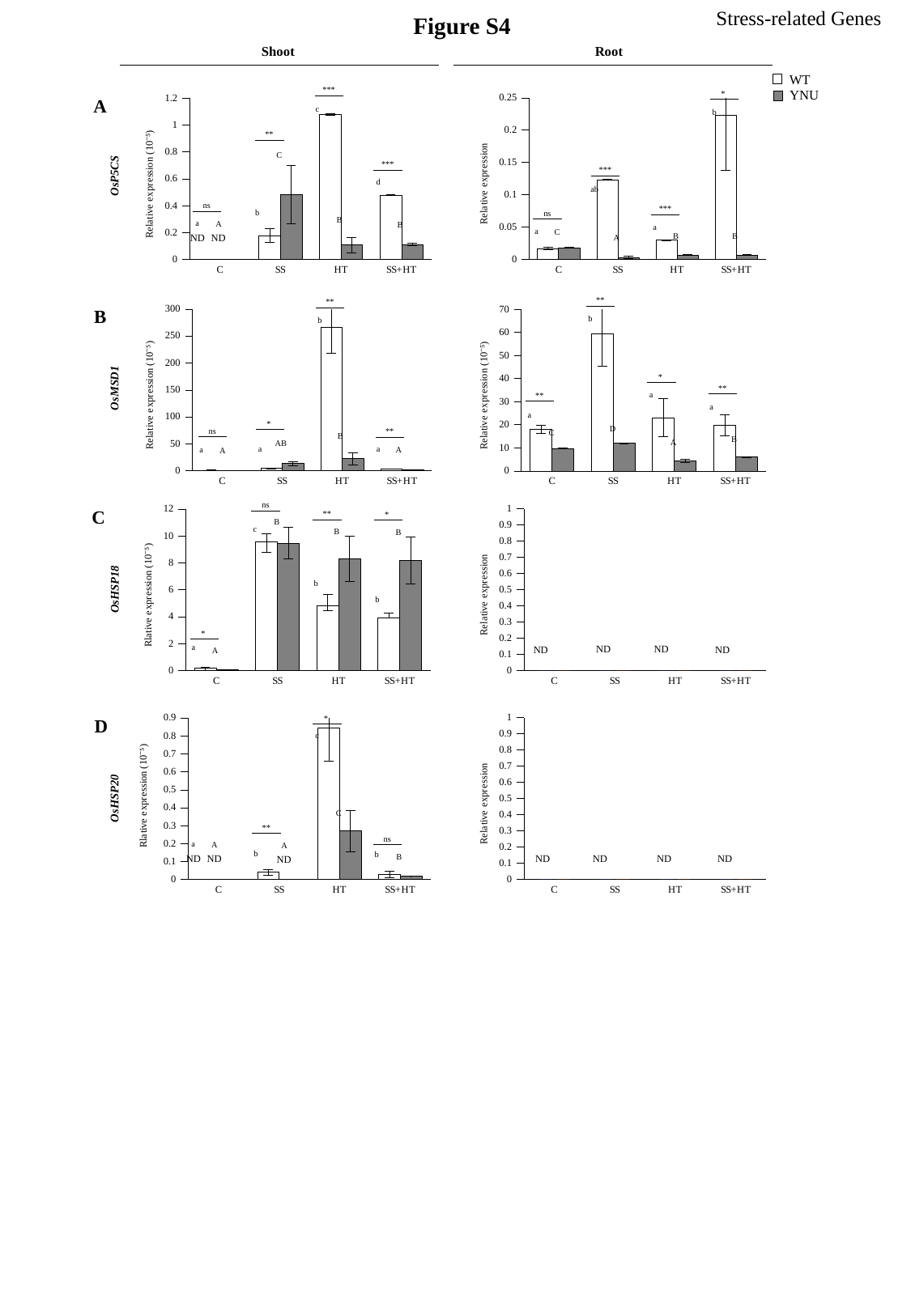

Stress-related Genes
Figure S4
Shoot
Root
WT
YNU
***
### Chart
| Category | Yukinkomai | YNU |
|---|---|---|
| C | 0.0 | 0.0 |
| SS | 0.17873024378555288 | 0.4847178003295353 |
| HT | 1.0781718841128136 | 0.1082311420042733 |
| SS+HT | 0.47853437168402785 | 0.11211215917454391 |OsP5CS
c
**
C
***
d
ns
b
B
a
A
B
ND
ND
*
### Chart
| Category | Yukinkomai | YNU |
|---|---|---|
| C | 0.016829945549784172 | 0.018099954772902785 |
| SS | 0.12321039228547583 | 0.003053791753715124 |
| HT | 0.030138995667498874 | 0.007086940850859383 |
| SS+HT | 0.22321708237741766 | 0.006910779323159359 |b
***
ab
***
ns
a
a
C
B
B
A
A
**
### Chart
| Category | Yukinkomai | YNU |
|---|---|---|
| C | 18.20162029155447 | 9.781838726523409 |
| SS | 59.29233974442561 | 11.99477111790245 |
| HT | 23.062912115386784 | 4.528328450553564 |
| SS+HT | 19.826029279525304 | 6.0604611678259275 |b
*
**
a
**
a
a
D
C
B
A
**
### Chart
| Category | Yukinkomai | YNU |
|---|---|---|
| C | 0.9725370110725832 | 0.15159001066628092 |
| SS | 4.172380513351963 | 13.236422859924895 |
| HT | 266.57569586492696 | 22.17701982181156 |
| SS+HT | 3.271983799069224 | 2.622053041638812 |OsMSD1
b
B
AB
a
a
a
A
A
*
ns
**
B
ns
### Chart
| Category | Yukinkomai | YNU |
|---|---|---|
| C | 0.19561814554782084 | 0.049953772582803375 |
| SS | 9.597787956364936 | 9.467930399672742 |
| HT | 4.842171964252125 | 8.299939657201413 |
| SS+HT | 3.9220623959306877 | 8.177915729989552 |OsHSP18
B
c
B
B
b
b
a
A
**
*
*
C
### Chart
| Category | Yukinkomai | YNU |
|---|---|---|
| C | 0.0 | 0.0 |
| SS | 0.0 | 0.0 |
| HT | 0.0 | 0.0 |
| SS+HT | 0.0 | 0.0 |ND
ND
ND
ND
*
### Chart
| Category | Yukinkomai | YNU |
|---|---|---|
| C | 0.0 | 0.0 |
| SS | 0.04008787251733947 | 0.0 |
| HT | 0.8434290829802755 | 0.2704365201148566 |
| SS+HT | 0.029841684769126308 | 0.018536506234137717 |OsHSP20
c
C
**
ns
a
A
A
b
b
B
ND
ND
ND
D
### Chart
| Category | Yukinkomai | YNU |
|---|---|---|
| C | 0.0 | 0.0 |
| SS | 0.0 | 0.0 |
| HT | 0.0 | 0.0 |
| SS+HT | 0.0 | 0.0 |ND
ND
ND
ND
