# Supplementary material for: Impact of Single and Combined Salinity and High-Temperature Stresses on Agro-Physiological, Biochemical, and Transcriptional Responses in Rice and Stress-Release
Source: Plants (Basel). 2022 Feb 12;11(4):501. doi: 10.3390/plants11040501 (PMC8876766; doi:10.3390/plants11040501)
Supplement: Supplementary file 1 [file plants-11-00501-s001.zip › Nahar et al_Supp Tables_Plants.pdf]

Supporting Information

Impact of single and combined salinity and high-temperature stresses on agro-physiological, biochemical, and transcriptional responses in rice and stress-release

Lutfun NAHAR[1,2], Murat AYCAN[3], Shigeru HANAMATA[3], Marouane BASLAM[3*], Toshiaki MITSUI[1,3*]

[1]Department of Life and Food Sciences, Graduate School of Science and Technology, Niigata University, Niigata, Japan.

[2]Department of Agricultural Botany, Sher-e-Bangla Agricultural University, Dhaka-1207, Bangladesh.

[3]Laboratory of Biochemistry, Faculty of Agriculture, Niigata University, Niigata, Japan.

Running Title: Specific responses/mechanisms to single and combined stresses in rice

***Author for correspondence:**

Toshiaki MITSUI, Ph.D., Prof.; Marouane BASLAM, Ph.D., Assist. Prof.

Laboratory of Biochemistry, Faculty of Agriculture, Niigata University, 8050 Ikarashi 2-no-cho, Nishi-ku, Niigata City, Niigata 950-2181, Japan.
Tel: +81-25-262-6641
Email: t.mitsui@agr.niigata-u.ac.jp; mbaslam@gs.niigata-u.ac.jp

Supporting Information

Table S1. Primer list

**Accession number**	**Gene name**	**Forward primer**	**Reverse primer**
Os12g0641100	*OsSOS1*	TTGCATCTACTTGGAATGGACAG	TACAGTAACCCAATCAGCAGGA
Os06g0606000	*OsSOS2*	ACGTTCGCCAAGGTCAAGTT	ATTGGGGTGTCTTACAATCTTCA
Os05g0534400	*OsSOS3*	CTCAGGGAGATGGTCTTGGC	CATGTTCCTTAGTGATGCCGG
Os01g0307500	*OsHKT1;5*	CTCTTCATGGCGGTCAACTC	CCGTTTGCTGGTGTTTGTC
Os03g0687000	*OsNPF2;4*	GGTGCTGTTCATGTCGTTCG	GCGGAGGTGAAGGTGAAGTA
Os07g0666900	*OsNHX1*	GGAAGAGCTCGCACTTATTCG	ATATCATTGTCCCGACGGCT
Os02g0658100	*OsTIP2;1*	ACATCACCATCCTCACCGG	TGACGATCTCCATCACGACG
Os02g0629200	*OsPIP2;3*	TGTTCATGGTTCACTTGGCC	CGCGGAAGGAGGAGGAAG
Os07g0448100	*OsPIP2;4*	GTACACCGTCTTCTCCGCC	CACTCCAGGCCTTGTTGTTG
Os04g0233400	*OsPIP2;5*	GTACACCGTCTTCTCCGCC	CCATGCCTTGCTGTTGTTGTA
Os05g0455500	*OsP5CS*	GACTGTTGGCACTGGAACTG	TGCTGTCATGCCTCCTCTAC
Os05g0323900	*OsMSD1*	AGGTGCTGCTTTACAAGGA	CCCAACAAAGGAACCAAGTT
Os01g0184100	*OsHSP18*	GAGGAGAAGGAGGAGTCGTG	TCGATGGTCTTGGGCTTCTT
Os03g0267000	*OsHSP20*	TCAGTTCATTCTTCTGTCGCC	ATTGGAGTAGGTTTCGCACA
Os01g0328400	*OsUBQ5*	ACCACTTCGACCGCCACTACT	ACGCCTAAGCCTGCTGGTT

Table S2. Loading values and percentage contribution of principal component (PC) variables for Fig. 6A.

Variables	PC1		PC2		PC3	
	Loading values	Contribution of variable (%)	Loading values	Contribution of variable (%)	Loading values	Contribution of variable (%)
SL	0.038	1.043	0.531	17.785	0.202	6.596
RL	-0.261	7.238	-0.095	3.173	0.399	13.013
SFW	-0.038	1.052	0.515	17.235	0.060	1.972
RFW	-0.194	5.379	0.256	8.555	0.114	3.722
SDM	-0.275	7.611	0.253	8.453	-0.283	9.233
RDM	-0.234	6.475	0.227	7.591	-0.126	4.099
Chla	-0.353	9.768	0.037	1.246	-0.038	1.252
Chlb	-0.317	8.766	-0.029	0.971	-0.039	1.261
ChlT	-0.323	8.944	0.080	2.666	-0.033	1.092
MDA	-0.256	7.089	-0.239	8.002	0.387	12.642
APX	-0.304	8.422	-0.133	4.456	0.061	1.976
CAT	-0.303	8.389	-0.075	2.495	-0.261	8.527
SOD	-0.186	5.155	-0.013	0.435	0.592	19.328
PRO	-0.324	8.970	0.103	3.431	-0.251	8.195
PTN	0.206	5.699	0.404	13.506	0.217	7.091
Standart deviation		2.744		1.682		1.292
Variability (%)		50.218		18.866		11.130
Cumulative (%)		50.218		69.084		80.215

Table S3. Loading values and percentage contribution of principal component (PC) variables for Fig. 6B.

Variables	PC1		PC2		PC3	
	Loading values	Contribution of variable (%)	Loading values	Contribution of variable (%)	Loading values	Contribution of variable (%)
SL	0.322	9.295	0.266	8.190	0.012	0.434
RL	0.214	6.179	-0.420	12.921	-0.247	9.167
SFW	0.309	8.939	0.243	7.488	0.057	2.112
RFW	0.302	8.738	0.182	5.602	-0.011	0.404
SDM	0.337	9.733	0.150	4.618	0.114	4.235
RDM	0.323	9.340	0.130	3.998	0.153	5.691
Chla	0.344	9.936	-0.088	2.719	-0.058	2.171
Chlb	0.320	9.236	-0.174	5.349	-0.065	2.416
ChlT	0.342	9.883	-0.113	3.467	0.094	3.480
MDA	0.119	3.431	-0.137	4.210	-0.518	19.214
APX	0.195	5.646	-0.439	13.490	0.264	9.804
CAT	-0.020	0.572	-0.109	3.347	0.573	21.258
SOD	-0.030	0.854	-0.078	2.407	0.462	17.155
PRO	0.059	1.691	-0.562	17.278	0.019	0.706
PTN	0.226	6.527	0.160	4.917	-0.047	1.754
Standart deviation		2.707		1.537		1.380
Variability (%)		48.859		15.753		12.705
Cumulative (%)		48.859		64.613		77.319

Table S4. Loading values and percentage contribution of principal component (PC) variables for Fig. 6C.

Variables	PC1		PC2		PC3	
	Loading values	Contribution of variable (%)	Loading values	Contribution of variable (%)	Loading values	Contribution of variable (%)
SL	0.298	8.002	0.233	6.989	0.095	2.722
RL	0.227	6.115	-0.067	2.015	-0.293	8.373
SFW	0.302	8.127	0.184	5.523	-0.249	7.106
RFW	0.280	7.525	0.142	4.250	-0.390	11.158
SDM	0.306	8.222	0.161	4.820	-0.257	7.342
RDM	0.291	7.809	0.133	3.969	-0.349	9.969
Chla	0.304	8.176	-0.183	5.479	0.222	6.346
Chlb	0.279	7.510	0.179	5.369	0.327	9.336
ChlT	0.303	8.149	0.035	1.060	0.338	9.676
MDA	0.265	7.133	-0.225	6.743	0.252	7.193
APX	0.263	7.076	-0.269	8.056	0.030	0.865
CAT	0.108	2.906	-0.461	13.817	-0.183	5.241
SOD	0.077	2.058	-0.489	14.631	-0.127	3.639
PRO	0.156	4.184	-0.421	12.599	0.044	1.258
PTN	0.261	7.009	0.156	4.680	0.342	9.778
Standart deviation		2.956		1.706		1.137
Variability (%)		58.253		19.415		8.62
Cumulative (%)		58.253		77.669		86.289

Table S5. Loading values and percentage contribution of principal component (PC) variables for Fig. 6D.

Variables	PC1		PC2		PC3	
	Loading values	Contribution of variable (%)	Loading values	Contribution of variable (%)	Loading values	Contribution of variable (%)
SL	0.287	7.781	0.113	3.693	0.263	7.512
RL	0.244	6.627	-0.225	7.372	-0.13	3.715
SFW	0.292	7.925	-0.038	1.252	0.359	10.259
RFW	0.297	8.071	-0.135	4.434	0.308	8.807
SDM	0.307	8.338	-0.068	2.231	0.297	8.500
RDM	0.301	8.185	-0.117	3.822	0.273	7.818
Chla	0.288	7.810	0.178	5.842	-0.243	6.940
Chlb	0.29	7.873	0.073	2.399	-0.08	2.303
ChlT	0.311	8.449	0.071	2.335	-0.117	3.356
MDA	0.246	6.689	-0.014	0.453	-0.514	14.696
APX	0.247	6.704	0.271	8.873	-0.147	4.199
CAT	0.095	2.583	-0.41	13.434	-0.241	6.906
SOD	-0.024	0.647	-0.534	17.520	-0.095	2.707
PRO	0.195	5.304	-0.461	15.126	-0.169	4.845
PTN	0.258	7.013	0.342	11.214	-0.26	7.435
Standart deviation		2.999		1.348		1.128
Variability (%)		59.995		12.119		8.497
Cumulative (%)		59.995		72.115		0.806
